# Safe engagement in physical activity through pre-exercise risk assessment: an observational study at a single facility over 16 years

**DOI:** 10.3389/fpubh.2025.1563385

**Published:** 2025-07-10

**Authors:** Akihiro Hirata, Yoshifusa Takao, Tomoaki Seto, Satoshi Kurose, Yoshinobu Saito, Shinji Sato, Shigeki Tsuzuku, Yuko Oguma

**Affiliations:** ^1^Research Fellow of Japan Society for the Promotion of Science, Tokyo, Japan; ^2^Sports Medicine Research Center, Keio University, Yokohama, Japan; ^3^LIFE Medical Fitness, Fujisawa, Japan; ^4^Health Science Center, Kansai Medical University, Hirakata, Japan; ^5^Faculty of Sport Management, Nippon Sport Science University, Yokohama, Japan; ^6^Graduate School of Physical Education, Health and Sport Studies, Nippon Sport Science University, Tokyo, Japan; ^7^Department of Sport and Medical Science, Teikyo University, Tokyo, Japan; ^8^Center for Student Success Research and Practice, The University of Osaka, Toyonaka, Japan; ^9^Graduate School of Health Management, Keio University, Fujisawa, Japan

**Keywords:** adverse events, health promotion facilities, medical screening, fitness club, swimming, squash

## Abstract

**Background:**

Regular and appropriate physical activity has health benefits; however, to ensure safety, a pre-exercise medical check based on health information is important. In this study, we aimed to clarify the relationship between risk classification by physicians at a health promotion facility in Japan and the occurrence of adverse events during facility use.

**Methods:**

We evaluated 3,571 individuals, excluding those with an unknown sex, age, medical assessment of exercise limitations, and facility usage status. Based on the results of the medical checkups conducted by a physician, the participants were divided into an exercise-prohibited group and an exercise-permitted group (exercise-permitted group, subdivided into non-restricted, orthopedic-restricted, internal medical-restricted, and combined-restricted groups). The risk of adverse events was examined.

**Results:**

The group in which exercise was prohibited comprised 72 participants, and that in which exercise was permitted comprised 1935, 612, 456, and 496 participants in the non-restricted, orthopedic-restricted, internal medical-restricted, and combined-restricted groups, respectively. Logistic regression analysis was performed on the four subgroups of the exercise-permitted group, and the odds ratios for adverse events adjusted for individual attributes were 1.04 [95% confidence interval (CI), 0.59–1.84; *p* = 0.89], 0.97 (95% CI, 0.53–1.78; *p* = 0.93), and 0.80 (95% CI, 0.42–1.54; *p* = 0.51) for the orthopedic-restricted, internal medical-restricted, and combined-restricted groups, respectively. A power analysis revealed that the study had a high level of power (0.99), based on a Cox–Snell *R*^2^ of 0.05 and a sample size of 3,499, indicating sufficient sensitivity to detect differences between groups.

**Conclusion:**

No significant difference in the odds of adverse events was found regardless of the presence or absence of exercise restrictions. Therefore, despite exercise-related risks, pre-exercise screening can help ensure that exercise is performed as safely as it is by individuals without such risks. However, further discussion is required regarding the necessity of screening for all exercise participants.

## 1 Introduction

Regular physical activity is a protective factor in the prevention and management of noncommunicable diseases (NCDs), including cardiovascular disease, type 2 diabetes, and breast and colon cancer (1). In Japan, the Physical Activity Guide for Health Promotion was published in January 2024 (2). This guide was revised from the one created in 2013 (3); it emphasizes “adjusting the intensity and amount of physical activity based on individual differences and starting with physical activities that are appropriate” (2). Additionally, it includes recommendations for strength training, precautions for individuals with chronic diseases, and information on the support environment for physical activity (2). As part of efforts to foster a social environment that promotes physical activity, it is important to improve the quality and number of physical activity instructors at exercise facilities and to strengthen physical activity guidance within medical institutions (2). Therefore, creating an environment in which a diverse range of individuals, including those with chronic diseases, can safely engage in physical activity is essential.

When engaging in a new type of physical activity, individuals need to manage their risks to ensure their safety. The risk of developing acute myocardial infarction increases when individuals without regular exercise routines suddenly engage in strenuous exercise (4). A large-scale survey of the Japanese population revealed that 71.3% of individuals have no exercise habits (i.e., do not exercise for at least 30 min at least twice a week for at least 1 year) (5). Individuals with chronic diseases—such as hypertension, diabetes, or dyslipidemia—need to take health precautions specific to their condition, for example, avoid high blood pressure or hypoglycemia before exercise and manage diabetes complications. Additionally, medications used for treatment should also be considered, for example, beta-blockers inhibit the heart rate from increasing effectively, so monitoring exercise intensity using heart rate is unsuitable. The prevalence of these diseases is higher in older than in younger individuals. In the Physical activity and exercise guide for health promotion 2023, it was reported that 60% of Japanese individuals aged ≥60 years attend healthcare facilities for some type of disease (2). Another report revealed that 80% of individuals aged ≥75 years had two or more diseases (6). A history of falls and medication use are risk factors for falls in older adults (7). In our previous study, we found that older adults in a population that voluntarily engaged in sports and physical activity in Japan often reported fall-related adverse events during activity (8).

Sports injuries often occur in athletes who engage in high-intensity physical activity (9–12). To prevent such injuries and sudden death during physical activity, detailed pre-exercise medical checks are performed (13). Additionally, our previous scoping review of adverse events during physical activity in the general population revealed that fatal accidents, cardiopulmonary arrest, and musculoskeletal injuries have been reported (14). Among the 67 articles included in the review, the authors of 13 (9 prospective and 4 retrospective studies) reported the frequency of adverse events, expressed in various units (e.g., per 1,000 person-hours, 1,000 person-days, or 1,000 person-exposures). For example, incidence rates per 1,000 person-hours were reported in studies concerning adult rugby, youth soccer, and other team sports, indicating that even among the general population, adverse events during physical activity are not negligible. In response to these risks, the American College of Sports Medicine (ACSM) proposes medical clearance for the general population before physical activity (15).

However, despite the widespread implementation of pre-exercise medical checkups for athletes, it is unclear whether these checkups (i.e., risk classification) can be used to enhance the safety of community-dwelling adults who participate in facility-based physical activity programs. Therefore, we aimed to clarify the relationship between health status-based exercise restrictions and adverse events among individuals who underwent medical checkups at an exercise facility. We hypothesized that individuals who received exercise restrictions based on medical checkups could engage in physical activity with a comparable risk of adverse events to those without such restrictions.

## 2 Materials and methods

### 2.1 Study setting and design

In this single-center observational study, data (April 2000–March 2022) were obtained from a health promotion facility (16) located in Fujisawa City, Kanagawa Prefecture, Japan. Health promotion facilities that meet certain standards and provide appropriate exercise guidance and health management are certified by the Ministry of Health, Labour and Welfare. They play a role in supporting users through the safe and effective promotion of healthy lifestyles and prevention of NCDs (17). This health promotion facility is a membership-based facility attached to a regional medical care support hospital (general hospital) with 330 beds. The exercise area is staffed by qualified health exercise programmers, who are trained in creating and delivering exercise programs with the aim of supporting exercise for health, preventing NCDs, and maintaining and improving health standards based on exercise physiology and medical knowledge (18).

When registering to use the facility, a physician conducts an interview with the user. The physician (specialized in orthopedics), who is qualified as a health sports physician through the Japan Medical Association, screens the user based on their health checkup data, current medical history, and past medical history, and decides whether they can exercise without restrictions, exercise with content- or intensity-related restrictions, or are prohibited from exercising (requiring a visit to a medical institution). This classification was made entirely based on the physician’s clinical judgment, without reference to any standardized internal guidelines. Restrictions on the exercise content are evaluated from two perspectives: restrictions owing to orthopedic problems and restrictions owing to internal medicine problems.

This study was conducted in accordance with the principles of the Declaration of Helsinki and the study protocol was approved by the Research Ethics Committee of the Sports Medicine Research Center, Keio University (Approval No.: 2022-06). The opt-out method was used to explain the study to the participants. A detailed notice was posted at the health promotion facility to inform users about the study’s purpose, methods, and data-handling procedures. This notice also explained that participation was voluntary and that users could freely decline participation without disadvantage. Participants who wished not to have their data included could opt out. This opt-out procedure ensured the protection of the participants’ rights and was approved by the research ethics committee.

This study conformed to the Strengthening the Reporting of Observational Studies in Epidemiology guidelines.

### 2.2 Participants and data

The participants were individuals who registered to use the health promotion facility between April 2000 and March 2022 (*n* = 5,137). We excluded individuals with an unknown sex or age, missing medical assessment data on exercise limitations, and who withdrew from the facility between April 2000 and June 2005. Owing to a change in the system at the facility, usage and adverse event information were unavailable between April 2000 and June 2005; therefore, we only included individuals with a usage history from July 2005 onward.

We evaluated data that had been recorded and stored as part of the facility’s regular operations, including age, height, weight, body mass index (BMI), body fat percentage, systolic blood pressure, diastolic blood pressure, resting electrocardiogram (ECG) findings at the time of registration, number of times the facility was used, period of enrollment, and information on adverse events that occurred at the facility. Owing to limitations in obtaining data at the beginning of the observation period, the number of times the facility was used, period of enrollment, and adverse events were recorded from July 2005 to March 2022.

### 2.3 Outcomes and measures

The outcome variable was the occurrence of adverse events during the use of the facility (1 for yes, 0 for no). Adverse events—including accidents, injuries, and the onset of illness—were recorded using the reporting formats routinely employed by the facility. These formats are implemented through institutional procedures that involve staff training and supervisory review, which facilitate consistency in the recorded information. The severity of adverse events was assessed by a review of each case to determine whether it required medical attention, such as a physician consultation or emergency care. Despite the limitations imposed by this classification, it was employed to differentiate between events that were deemed non-serious and those that were medically confirmed or potentially serious. The exposure variable was the presence or absence of exercise restrictions as classified by the physician, and it was divided into four groups: non-restricted group, orthopedic-restricted group, internal medical-restricted group, and combined-restricted group. The following personal attributes were used: sex (male, female), age (years), height (cm), weight (kg), BMI (kg/m^2^), body fat percentage (%), systolic blood pressure, diastolic blood pressure (mmHg), resting ECG result (normal, 0; abnormal, 1), number of times the facility was used (days), and length of enrollment (years).

Self-reported sex and age were obtained from a medical questionnaire. Height, weight, body fat percentage, and resting blood pressure were measured. Resting ECG results were coded as “normal = 0” and “abnormal = 1” based on medical judgment documented in the reports brought by the participants. These ECG reports had been issued by medical institutions, and the interpretation (e.g., normal, requires follow-up, or requires medical attention) was made by attending physicians. The criterion for “abnormal” was any indication of abnormality (e.g., arrhythmia, ischemic changes) noted by the physician.

### 2.4 Statistical analyses

The participants were divided into four groups based on the results of the medical checkup conducted by the physician before they started using the facility: the exercise-prohibited and exercise-permitted group, which was further subdivided into the non-restricted group, orthopedic-restricted group, internal medical-restricted group, and combined-restricted group. Continuous variables are presented as medians (interquartile ranges) and categorical variables as *n* (%). Comparisons were made among the four exercise-permitted groups for each variable. For continuous variables, the Kruskal–Wallis and Mann–Whitney U tests with Bonferroni correction were used. For categorical variables, the *χ*^2^ test and residual analysis (significant at adjusted residuals of ≥1.96) were used.

Reported adverse events were categorized using an inductive thematic analysis. One researcher initially reviewed and coded the free-text descriptions of the event content and causes. The coding results were subsequently audited and confirmed by multiple other researchers to ensure consistency and validity. Examples included musculoskeletal pain and general fatigue during exercise.

Logistic regression analysis was used to estimate the risk of adverse events with or without exercise restriction. The dependent variable was adverse events (1 for present, 0 for absent), and the independent variable was exercise restriction (1 for the non-restricted group, 2 for the orthopedic-restricted group, 3 for the internal medical-restricted group, 4 for the combined-restricted group). In the logistic regression analysis, the “non-restricted” group was used as the reference category. This group consisted of individuals who, based on physician assessment, had no medical restrictions pertaining to exercise participation. Using this group as a reference allowed us to evaluate the increased likelihood of adverse events in participants with medical restrictions on exercise. The data were adjusted for individual attributes (sex, age, BMI, body fat percentage, systolic blood pressure, diastolic blood pressure, resting ECG findings, frequency of facility use, and length of enrollment). The missing data rate in this study was <5% (129/3499 cases, 3.7%), and a complete case analysis was performed in accordance with the Treatment And Reporting of Missing data in Observational Studies framework (19). We conducted sensitivity analyses using weight-based single imputation, a method where missing values are imputed once based on weighted estimates considering relevant participant characteristics, which revealed no substantial difference in results. To evaluate the power of the logistic regression analysis, we performed a *post-hoc* power analysis using G*Power 3.1.7.1. The effect size was the Cox–Snell *R*^2^ obtained from the logistic regression analysis (0.05), and the sample size was the number of participants used in this analysis.

All statistical tests were two-tailed, and statistical significance was set at *p* < 0.05. SPSS Statistics 29.0 (IBM Corp., NY, USA) was used for statistical analyses.

## 3 Results

### 3.1 Participant characteristics

**Figure 1:**
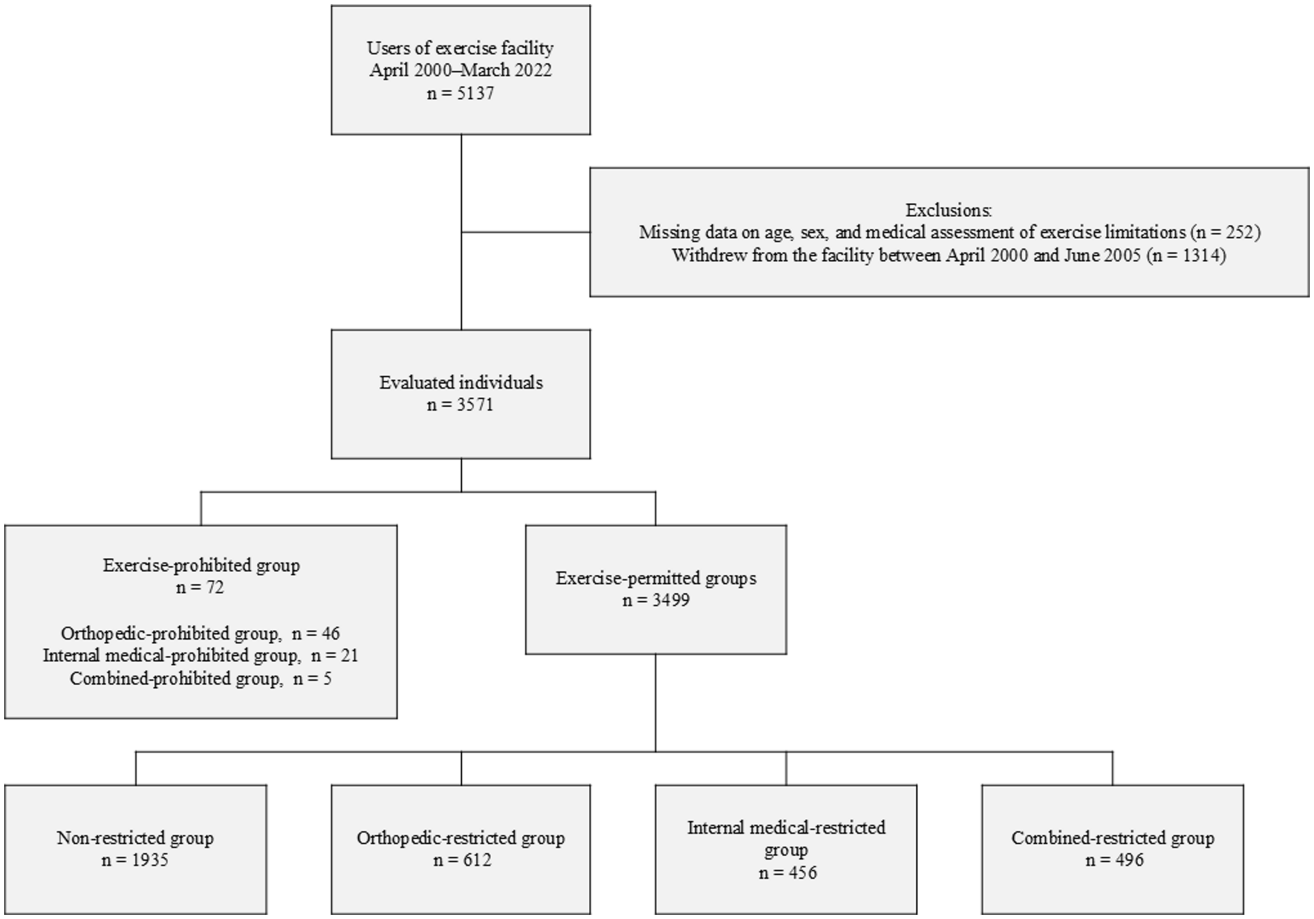
Flow diagram of participant selection and grouping.

Between April 2000 and March 2022, 5,137 individuals were registered at the facility. Of these, 3,571 were eligible for this study after excluding those who met the exclusion criteria. Among eligible participants, 72 were placed in the exercise-prohibited group based on physician assessment, and 3,499 started exercising in the exercise-permitted group. The exercise-permitted group was further classified into four subgroups: non-restricted (*n* = 1935), orthopedic-restricted (*n* = 612), internal medical-restricted (*n* = 456), and combined-restricted (*n* = 496) (Figure 1).

The median age of the exercise-prohibited group was 59 (IQR 47.3–70) years; 46 were restricted due to orthopedic problems, 21 due to internal medical problems, and 5 due to both (Table 1). The overall proportion of women was 58.1%. Among the exercise-permitted subgroups, the orthopedic-restricted group had a significantly lower proportion of women, whereas the internal medical-restricted group had a higher proportion (*p* < 0.001). The median age was lowest in the non-restricted group [39 (29–53) years] and highest in the combined-restricted group [62 (52–69) years]. The combined-restricted group also showed significantly higher BMI, body fat percentage, systolic and diastolic blood pressure, and abnormal resting ECG findings compared to the non-restricted group. All differences among groups were statistically significant (Kruskal–Wallis test, *p* < 0.001) (Table 2). These characteristics likely reflect the underlying medical conditions—such as NCDs or osteoarthritis—that prompted the exercise restrictions. Clinically, as age increases, the prevalence of orthopedic conditions such as osteoarthritis, hypertension, and pathological changes indicated by abnormal ECG findings also rises. Consequently, the management of risk during exercise assumes paramount importance for these individuals. Consequently, the combined-restricted group in this study represents a high-risk population regarding exercise.

**Table 1:** Personal attributes of the exercise-prohibited group.

Participant characteristics	Orthopedic-prohibited group		Internal medical-prohibited group		Combined-prohibited group		Overall	
	*n* = 46		*n* = 21		*n* = 5		*n* = 72	
Sex, female, *n* (%)	18	(39.1)	16	(76.2)	2	(40.0)	36	(50.0)
Age (years)	55.5	(46.8–68.3)	67.0	(45.0–77.0)	70.0	(59.0–78.5)	59.0	(47.3–70.0)
Height (cm)	165.5	(159.0–170.0)	155.0	(149.0–159.0)	158.0	(152.5–169.0)	160.5	(154.0–169.0)
Weight (kg)	66.5	(59.0–81.5)	55	(49.5–66.5)	51.5	(49.5–64.0)	62.0	(54.0–77.0)
BMI (kg/m^2^)	26.6	(22.4–29.2)	23.5	(21.25–26.0)	21.3	(19.0–33.5)	24.8	(21.8–28.0)
Body fat percentage (%)	28.0	(24.0–34.5)	27.0	(25.0–34.5)	23.0	(19.5–33.5)	27.0	(24.0–34.0)
Missing, *n* (%)	1	(2.2)					1	(1.4)
Systolic BP (mmHg)	144.0	(127.8–160.5)	142.0	(128.5–149.0)	136.0	(124.5–184.5)	143.0	(127.3–159.5)
Diastolic BP (mmHg)	83.0	(73.3–100.0)	79.0	(71.5–84.5)	84.0	(69.5–103.5)	81.0	(71.5–93.0)
Resting ECG, abnormal, *n* (%)	10	(21.7)	3	(14.3)	3	(60.0)	16	(22.2)
Missing, *n* (%)	12	(26.1)	1	(4.8)	1	(20.0)	14	(19.4)

Data are presented as median (interquartile range). BMI, body mass index; BP, blood pressure; ECG, electrocardiogram.

**Table 2:** Personal attributes of each group that started exercising.

Participant characteristics	Non-restricted group		Orthopedic-restricted group		Internal medical-restricted group		Combined-restricted group		Overall		*p*-Value
	*n* = 1935		*n* = 612		*n* = 456		*n* = 496		*n* = 3,499		
Sex, female, *n* (%)	1,107	(57.2)	294	(48.0)^*^	340	(74.6)^**^	303	(61.1)	2044	(58.4)	<0.001
Age (years)	39	(29–53)^b,c,d^	56	(46–64)^a,d^	56	(44–63)^a,d^	62	(52–69)^a,b,c^	49	(34–60)	<0.001
Height (cm)	163	(157–170)^c,d^	162	(156–169)^c,d^	158	(153–164)^a,b,d^	159	(153–166)^a,b,c^	162	(156–169)	<0.001
Missing, *n* (%)	35	(1.8)	5	(0.8)	6	(1.3)	4	(0.8)	50	(1.4)	
Weight (kg)	61	(52.0–70.0)^b,c,d^	65	(56.0–75.8)^a,c^	58	(52.8–66.3)^a,b,d^	64	(56.0–73.0)^a,c^	62	(53.0–71.0)	<0.001
Missing, *n* (%)	34	(1.8)	4	(0.7)	6	(1.3)	1	(0.2)	45	(1.3)	
BMI (kg/m^2^)	22.8	(20.7–25.2)^b,d^	25	(22.3–27.6)^a,c^	23.2	(21.2–25.5)^b,d^	25	(22.5–28.2)^a,c^	23.4	(21.2–26.1)	<0.001
Missing, *n* (%)	37	(1.9)	5	(0.8)	6	(1.3)	4	(0.8)	52	(1.5)	
Body fat percentage (%)	26	(22–30)^b,c,d^	28	(23–33)^a^	28.5	(24–33)^a^	30	(24–35)^a^	27	(22–32)	<0.001
Missing, *n* (%)	66	(3.4)	11	(1.8)	8	(1.8)	7	(1.4)	92	(2.6)	
Systolic BP (mmHg)	122	(112.0–133.0)^b,c,d^	138.5	(124.0–152.0)^a,c^	127	(118.0–138.0)^a,b,d^	138	(125.0–149.0)^a,c^	127	(116.0–140.0)	<0.001
Missing, *n* (%)	44	(2.3)	2	(0.3)	7	(1.5)	4	(0.8)	57	(1.6)	
Diastolic BP (mmHg)	73	(65.0–80.0)^b,c,d^	81	(72.0–91.0)^a,c^	75	(68.0–82.0)^a,b,d^	80	(71.0–88.8)^a,c^	76	(68.0–84.0)	<0.001
Missing, *n* (%)	44	(2.3)	2	(0.3)	7	(1.5)	4	(0.8)	57	(1.6)	
Resting ECG, abnormal, *n* (%)	142	(7.3)^*^	135	(22.1)^**^	54	(11.8)	146	(29.4)^**^	477	(13.6)	<0.001
Missing, *n* (%)	73	(3.8)	14	(1.3)	10	(2.2)	8	(1.6)	105	(3.0)	
Years of facility use (years)	2	(1–5)^b,c,d^	2	(1–6)^a,c^	4	(1–9)^a,b^	3	(1–7)^a^	2	(1–6)	<0.001
Frequency of use (days)	37	(7–210)^b,c,d^	59	(9–341)^a^	81	(16–426)^a^	62	(11–312)^a^	50	(8–272)	<0.001

Data are presented as median (interquartile range). BMI, body mass index; BP, blood pressure; ECG, electrocardiogram.

^*^Adjusted residual analysis was performed after the *χ*^2^ analysis; this item was significantly less frequent. ^**^This item was significantly more frequent (*p* < 0.05).

*p*-Values in the right column represent the results of the Kruskal–Wallis test for continuous variables.

^a^vs. non-restricted group, ^b^vs. Orthopedic-restricted group, ^c^vs. Medical-restricted group, ^d^vs. combined-restricted group, analyzed using Mann–Whitney’s U test with Bonferroni correction, *p* < 0.05.

### 3.2 Adverse events

The total number of days of facility use was 1,150,709 person-days. A total of 136 cases of adverse events were reported during the 16 years and 9 months of observation. Reported events were classified into the following categories: musculoskeletal pain (*n* = 50), general fatigue (*n* = 44), contusion (*n* = 23), and wounds (*n* = 19). Musculoskeletal pain was most commonly associated with muscle injury (*n* = 14) and fracture (*n* = 13). General fatigue was most commonly reported in the form of dizziness (*n* = 11). The overall adverse event rate was 0.12 events per 1,000 person-days of facility use (exposures). The incidence rates of each adverse event category were as follows: musculoskeletal pain, 0.04 cases per 1,000 person-days; general fatigue, 0.04 cases per 1,000 person-days; contusion, 0.02 cases per 1,000 person-days; and wounds, 0.02 cases per 1,000 person-days (Table 3).

**Table 3:** Incidence of adverse events during the observation period.

Adverse event category	Cases	Incidence per 1,000 person-years	Incidence per 1,000 person-days
Adverse events	136	8.75	0.12
Musculoskeletal pain	50	3.22	0.04
General fatigue	44	2.83	0.04
Contusion	23	1.48	0.02
Wound	19	1.22	0.02
Total participants	3,499		
Total person-years		15,538	
Total person-days			1,150,709

There were four cases of cerebral infarction, three of myocardial infarction, and three of impaired consciousness, which were considered to likely be of high urgency. Although not all participants sought medical attention, medical institution visits varied by event type and sex; for example, medical visits were reported in 60.0% of musculoskeletal pain cases (52.2% male, 66.7% female), 65.9% of general fatigue cases (85.7% male, 56.7% female), 30.4% of contusions (11.1% male, 42.9% female), and 36.8% of wounds (33.3% male, 42.9% female). Cases requiring precise medical evaluation, such as fractures, cerebral infarction, and myocardial infarction, were diagnosed based on medical reports from medical institutions. General fatigue and contusion occurred most frequently in the swimming pools, whereas musculoskeletal pain occurred most frequently on the squash courts. Additionally, musculoskeletal pain and general fatigue were most frequently reported during or immediately after exercise, whereas contusions were most frequently reported falls (Table 4). No fatal accidents were reported.

**Table 4:** Description of reported adverse events.

Category	Male				Female				Overall			
	*n* = 58				*n* = 78				*n* = 136			
	Musculoskeletal pain	General fatigue	Contusion	Wound	Musculoskeletal pain	General fatigue	Contusion	Wound	Musculoskeletal pain	General fatigue	Contusion	Wound
	*n* = 23	*n* = 14	*n* = 9	*n* = 12	*n* = 27	*n* = 30	*n* = 14	*n* = 7	*n* = 50	*n* = 44	*n* = 23	*n* = 19
Classification of exercise restrictions												
Non-restricted group	15 (65.2)	5 (35.7)	3 (33.3)	7 (58.3)	16 (59.3)	16 (53.3)	6 (42.9)	2 (28.6)	31 (62.0)	21 (47.7)	9 (39.1)	9 (47.4)
Orthopedic-restricted group	5 (21.7)	7 (50.0)	4 (44.4)	2 (16.7)	3 (11.1)	3 (10.0)	3 (21.4)		8 (16.0)	10 (22.7)	7 (30.4)	2 (10.5)
Internal medical-restricted group	1 (4.3)	1 (7.1)		2 (16.7)	4 (14.8)	7 (23.3)	3 (21.4)	2 (28.6)	5 (10.0)	8 (18.2)	3 (13)	4 (21.1)
Combined-restricted group	2 (8.7)	1 (7.1)	2 (22.2)	1 (8.3)	4 (14.8)	4 (13.3)	2 (14.3)	3 (42.9)	6 (12.0)	5 (11.4)	4 (17.4)	4 (21.1)
Age group												
20s	1 (4.3)				3 (11.1)	3 (10)	2 (14.3)		4 (8.0)	3 (6.8)	2 (8.7)	
30s	3 (13)	1 (7.1)	1 (11.1)	1 (8.3)	2 (7.4)	1 (3.3)	1 (7.1)		5 (10.0)	2 (4.5)	2 (8.7)	1 (5.3)
40s	7 (30.4)		1 (11.1)	1 (8.3)	3 (11.1)	3 (10)			10 (20.0)	3 (6.8)	1 (4.3)	1 (5.3)
50s	4 (17.4)	2 (14.3)		2 (16.7)	5 (18.5)	6 (20.0)	1 (7.1)	1 (14.3)	9 (18.0)	8 (18.2)	1 (4.3)	3 (15.8)
60s	4 (17.4)	5 (35.7)	5 (55.6)	3 (25.0)	10 (37.0)	5 (16.7)	5 (35.7)	3 (42.9)	14 (28.0)	10 (22.7)	10 (43.5)	6 (31.6)
70s	3 (13.0)	6 (42.9)	2 (22.2)	4 (33.3)	2 (7.4)	7 (23.3)	4 (28.6)	1 (14.3)	5 (10.0)	13 (29.5)	6 (26.1)	5 (26.3)
80s	1 (4.3)			1 (8.3)	2 (7.4)	5 (16.7)	1 (7.1)	2 (28.6)	3 (6.0)	5 (11.4)	1 (4.3)	3 (15.8)
Location of occurrence												
Training gym	5 (21.7)	5 (35.7)	4 (44.4)	2 (16.7)	4 (14.8)	7 (23.3)	2 (14.3)		9 (18.0)	12 (27.3)	6 (26.1)	2 (10.5)
Studios					5 (18.5)	2 (6.7)			5 (10.0)	2 (4.5)		
Swimming pool	6 (26.1)	8 (57.1)	2 (22.2)	3 (25.0)	3 (11.1)	6 (20.0)	8 (57.1)	2 (28.6)	9 (18.0)	14 (31.8)	10 (43.5)	5 (26.3)
Squash courts	11 (47.8)	1 (7.1)	3 (33.3)	5 (41.7)	9 (33.3)	1 (3.3)	4 (28.6)	1 (14.3)	20 (40.0)	2 (4.5)	7 (30.4)	6 (31.6)
Front desk						6 (20.0)				6 (13.6)		
Changing rooms					3 (11.1)	7 (23.3)		3 (42.9)	3 (6.0)	7 (15.9)		3 (15.8)
Bathrooms				2 (16.7)	1 (3.7)	1 (3.3)		1 (14.3)	1 (2.0)	1 (2.3)		3 (15.8)
Others	1 (4.3)				2 (7.4)				3 (6.0)			
Cause/circumstances of occurrence												
During/immediately after exercise	18 (78.3)	7 (50.0)		1 (8.3)	15 (55.6)	15 (50.0)		1 (14.3)	33 (66.0)	22 (50.0)		2 (10.5)
Fall	4 (17.4)	1 (7.1)	3 (33.3)	3 (25.0)	10 (37.0)	1 (3.3)	9 (64.3)	1 (14.3)	14 (28.0)	2 (4.5)	12 (52.2)	4 (21.1)
Collision (with object)		1 (7.1)	3 (33.3)	3 (25.0)	1 (3.7)		2 (14.3)	4 (57.1)	1 (2.0)	1 (2.3)	5 (21.7)	7 (36.8)
Collision (with person)			3 (33.3)	1 (8.3)	1 (3.7)		3 (21.4)		1 (2.0)		6 (26.1)	1 (5.3)
Damage to equipment				3 (25.0)				1 (14.3)				4 (21.1)
Sauna/bathing		2 (14.3)		1 (8.3)		8 (26.7)				10 (22.7)		1 (5.3)
Others	1 (4.3)	3 (21.4)				6 (20.0)			1 (2.0)	9 (20.5)		
Was a medical institution visited?												
Yes	12 (52.2)	12 (85.7)	1 (11.1)	4 (33.3)	18 (66.7)	17 (56.7)	6 (42.9)	3 (42.9)	30 (60.0)	29 (65.9)	7 (30.4)	7 (36.8)
No	11 (47.8)	2 (14.3)	8 (88.9)	8 (66.7)	9 (33.3)	13 (43.3)	8 (57.1)	4 (57.1)	20 (40.0)	15 (34.1)	16 (69.6)	12 (63.2)
Total	24 (100)	14 (100)	9 (100)	12 (100)	27 (100)	3 (100)	14 (100)	8 (100)	51 (100)	44 (100)	23 (100)	2 (100)

Data are presented as *n* (%). The blank cells indicate that there were no reports: 0 (0%).

### 3.3 Association between exercise restriction categories and adverse event occurrence

We assessed the odds of adverse events owing to exercise restriction. Using the occurrence of adverse events in the non-restricted group as reference, we confirmed the adjusted odds of occurrence in the orthopedic-restricted, internal medical-restricted, and combined-restricted groups. After adjusting for individual attributes (sex, age, BMI, body fat percentage, blood pressure, resting ECG findings, frequency of facility use, and length of enrollment), the odds of adverse events were 1.04 [95% confidence interval (CI), 0.59–1.84; *p* = 0.89] for the orthopedic-restricted group, 0.97 (95% CI, 0.53–1.78; *p* = 0.93) for the internal medical-restricted group, and 0.80 (95% CI, 0.42–1.54; *p* = 0.51) for the combined-restricted group, compared with the non-restricted group (Table 5). None of the comparisons reached statistical significance with no exercise-restriction category being significantly associated with increased or decreased odds of adverse events compared to the non-restricted group. A *post-hoc* power analysis, based on a Cox–Snell R^2^ of 0.05 (from SPSS) and a sample size of 3,499, revealed a statistical power of 0.99, indicating a very high level of power. Despite the large sample size, no statistically significant differences were found, which supports the credibility of our null findings. This suggests that, under medically supervised conditions with tailored guidance, individuals with orthopedic or internal medical restrictions did not experience significantly elevated risks of adverse events.

**Table 5:** Odds ratios for adverse events by classification of exercise restriction.

Group	*n*	Crude^a^				Model 1^b^				Model 2^c^			
		OR	95% CI		*p* value	OR	95% CI		*p* value	OR	95% CI		*p* value
Non-restricted group	1935	1	Reference			1	Reference			1	Reference		
Orthopedic-restricted group	612	1.43	0.88	2.31	0.15	0.95	0.57	1.59	0.86	1.04	0.59	1.84	0.89
Internal medical-restricted group	456	1.29	0.74	2.25	0.37	0.88	0.50	1.58	0.68	0.97	0.53	1.78	0.93
Combined-restricted group	496	1.17	0.67	2.04	0.57	0.65	0.36	1.19	0.17	0.80	0.42	1.54	0.51

OR, odds ratio; CI, confidence interval.

a Crude: unadjusted model.

b Model 1: adjusted for age and sex.

c Model 2: additionally adjusted for body mass index, body fat percentage, systolic blood pressure, diastolic blood pressure, resting electrocardiogram findings, frequency of use (days), and length of enrollment.

## 4 Discussion

In this study, we aimed to clarify the relationship between restrictions on exercise content according to the results of a pre-exercise medical checkup and the occurrence of adverse events. Based on the medical checkup, 2% of the participants were prohibited from exercising, while 54% received no exercise restrictions. Age, BMI, body fat percentage, blood pressure, and the percentage of abnormal findings on the resting ECG were higher in the group with restrictions due to orthopedic and internal-medicine problems than in the group with no restrictions. The odds of adverse events owing to exercise restriction were not statistically significant, and the power of this analysis was sufficient. A total of 136 adverse events were reported during the observation period of 16 years and 9 months. The frequencies of adverse events were 8.75 per 1,000 person-years (length of enrollment) and 0.12 per 1,000 person-days (days of facility use).

According to the National Health and Nutrition Survey, the average values for Japanese citizens of the same age as the participants in this study (40–49 years) are as follows: BMI (24.7 kg/m^2^ for men and 22.3 kg/m^2^ for women), systolic blood pressure (125.4 mmHg for men and 113.7 mmHg for women), and diastolic blood pressure (80.6 mmHg for men and 70.9 mmHg for women) (20). Therefore, the characteristics of the target population did not significantly differ from those of the Japanese population. This similarity enhances the generalizability of our findings to the broader Japanese middle-aged adult population. These findings may be generalizable to other regions and facilities; however, differences in facility structure, staffing, and safety protocols may limit direct comparison and should be considered.

The incidence rate of adverse events found in the present study (0.12 cases/1,000 person-days) is comparable with those found in our previous study that was conducted in public training rooms (0.07 cases/1,000 person-days) (21) and another study on general physical activities (ranging from 0.015 to 0.118 cases/1,000 person-days) (22). However, differences in study populations (e.g., age, comorbidities), physical activity intensity (e.g., squash vs. walking), and adverse event reporting systems should be considered when interpreting these rates. The relatively higher rate in the present study may reflect both the inclusion of moderate-to-high intensity activities and the use of a structured reporting system within a medically supervised facility. Overall, while adverse events were not negligible, their frequency appears similar to or slightly higher than those found in similar contexts (21, 22), which suggests that appropriate safety measures can allow even high-risk individuals to engage in physical activity relatively safely. Additionally, in the facility used in this study, health consultations are conducted. Facility staff with specialized certifications also provide exercise guidance; therefore, individuals can safely undertake physical activity even if restrictions are necessary because of health issues. Notably, many adverse events occurred among participants without medical restrictions, likely due to engagement in more intense activities such as squash. However, in terms of generalizability, the need for a physician to conduct a medical checkup for all exercise participants in the same manner as in this study should be discussed.

Approximately 50% of our participants had no exercise restrictions. However, we found no difference in the odds of adverse events between the groups that required exercise restriction and the group that did not. Additionally, no fatal accidents were reported during the observation period. This may be attributed to the participants and facility staff having understood the risks through the pre-exercise medical checkups. The ACSM’s pre-exercise medical clearance is intended to identify participants who are at risk of sudden death or acute myocardial infarction while preventing excessive physical activity and unnecessary referrals to physicians (15).

The differences in the attributes of each group should be considered. As shown in Table 2, there were statistically significant differences between the groups for many personal attribute variables. The group with no exercise restrictions was a younger, healthier group. Although the intensity of the exercise undertaken in this study is unknown, many adverse events occurred on the squash court. It is expected that adverse events such as musculoskeletal pain occur when individuals in relatively good health—who can play squash—engage in relatively high-intensity physical activity. Parkkari et al. (23) reported that more adverse events during physical activity occurred in sports such as soccer and tennis than in walking and golf. Therefore, it is necessary to prepare for sports injuries and other problems during physical activities with higher exercise intensities. However, even activities associated with a low incidence of adverse events, such as walking, require appropriate precautions when performed in large groups, as the absolute number of adverse events per session may increase.

We found that the most common adverse events were musculoskeletal pain and general fatigue. Musculoskeletal pain occurred more frequently among participants without exercise restrictions, likely reflecting higher-intensity activities such as squash or gym training. General fatigue was more common among individuals in their 60s–70s and occurred in all areas of the facility. Most cases occurred during or immediately after exercise; however, notably, they also occurred during sauna and bathing. Musculoskeletal pain and contusion occurred because of falls. Most individuals with musculoskeletal pain and general fatigue visited a medical institution, whereas many with contusions and wounds did not.

Therefore, we propose the following three recommendations for sports facilities:

1. Regarding the prevention of and response to musculoskeletal pain in active participants, it is necessary to prepare responses, such as icing and immobilization, and equipment necessary for transport to a medical institution.
2. As general fatigue can occur anywhere in the facility, the staff involved in delivering exercise programs may not always be able to respond. Therefore, it is important to formulate and implement emergency response plans (24) to enable timely responses, regardless of where or by whom.
3. Measures to prevent falls should be implemented. Many cases of musculoskeletal pain and contusions are triggered by falls, which are associated with age, sex (female), fear of falling, a history of falls, poor eyesight, depression, and balance dysfunction (6). Consideration must also be given to environmental factors, such as floor conditions, pathways, and clothing and shoes worn (3, 25).

Therefore, we propose the following three recommendations for sports facilities: first, regarding the prevention of and response to musculoskeletal pain in active participants, it is necessary to prepare responses, such as icing and immobilization, and equipment necessary for transport to a medical institution. Second, as general fatigue can occur anywhere in the facility, the staff involved in delivering exercise programs may not always be able to respond. Therefore, it is important to formulate and implement emergency response plans (24) to enable timely responses, regardless of where or by whom. Third, measures to prevent falls should be implemented. Many cases of musculoskeletal pain and contusions are triggered by falls, which are associated with age, sex (female), fear of falling, a history of falls, poor eyesight, depression, and balance dysfunction (6). Consideration must also be given to environmental factors, such as floor conditions, pathways, and clothing and shoes worn (3, 25).

This study has various strengths. First, we used data from a long period, extending from 2005 to 2022 (16 years and 9 months). Second, as the medical checkups were conducted by the same physician, the evaluations were based on consistent criteria throughout the observation period. Third, the sample size was relatively large and sufficient based on the results of the *post-hoc* power analysis. Furthermore, there was no difference in the odds ratio of adverse events owing to exercise restrictions. This study provides useful findings for exercise facilities to support the promotion of physical activity.

### 4.1 Limitations

Because this was a retrospective study, all the necessary information could not be collected. For example, there were missing data for the first 6 years of the observation period (frequency of use of the facility, length of enrollment, adverse events). Furthermore, the content and intensity of the physical activities performed by the participants were not available, and information on their medication status and underlying health conditions was also lacking. Without information on the type and intensity of exercise performed, it is difficult to attribute adverse events to participant health status versus activity characteristics. Second, the participants were from a single facility, and the pre-exercise medical checkup was conducted by a single physician. Consequently, there are issues with objective validity and generalizability. However, the use of a single physician ensured consistent judgement standards throughout the study period, which may be considered a methodological strength.

## 5 Conclusion

The results of this study revealed no significant differences in the odds of adverse events according to exercise restriction status. One possible explanation is that medical checkups prior to exercise, followed by appropriate risk management, may have contributed to safety. Additionally, the presence of qualified staff at the facility may have supported safer physical activity. However, we did not directly assess the effectiveness of such measures, and the necessity of medical checkups for all participants remains to be determined. These findings suggest a potential association between structured pre-exercise screening, professional support, and safe physical activity. In particular, they indicate that even individuals with exercise-related restrictions may engage in physical activities safely when proper supervision and support are provided. Nevertheless, further research is needed to confirm the generalizability of these findings.

## Data Availability

The raw data supporting the conclusions of this article will be made available by the authors, without undue reservation.
